# Effects of exposure to glyphosate on oxidative stress, inflammation, and lung function in maize farmers, Northern Thailand

**DOI:** 10.1186/s12889-022-13696-7

**Published:** 2022-07-14

**Authors:** Sutthinee Sidthilaw, Ratana Sapbamrer, Chaicharn Pothirat, Klintean Wunnapuk, Supakit Khacha-ananda

**Affiliations:** 1 Department of Community Medicine, Faculty of Medicine, Chiang Mai University, Chiang Mai, 50200 Thailand; 2 Division of Pulmonary, Critical Care and Allergy, Department of Internal Medicine, Faculty of Medicine, Chiang Mai University, Chiang Mai, 50200 Thailand; 3 Department of Forensic Medicine, Faculty of Medicine, Chiang Mai University, Chiang Mai, 50200 Thailand

**Keywords:** Glyphosate, Oxidative stress, Inflammation, Lung function

## Abstract

**Background:**

Glyphosate is a herbicide which is commonly used in agricultural areas. However, previous studies on glyphosate exposure in farmers and their health are still scarce.

**Methods:**

A longitudinal pre-post study was performed among maize farmers. Information from questionnaires, urine and blood samples, and lung function were collected a day before and a day after glyphosate application in the morning. The urine samples were analyzed using liquid chromatography-tandem mass spectrometry to detect glyphosate levels. Serum samples were analyzed to detect malondialdehyde (MDA), glutathione (GHS), and C-reactive protein (CRP) levels using thiobarbituric acid, dithiobisnitrobenzoic acid, and nephelometry, respectively. Lung function performances were measured using a spirometer.

**Results:**

A total of 180 maize farmers met the study inclusion criteria. After glyphosate application, it was found that increased urinary glyphosate levels contributed to increased serum MDA (β = 0.024, 95% CI = 0.000, 0.0047) and decreased serum GHS (β = -0.022, 95% CI = -0.037, -0.007), FEV_1_ (β = -0.134, 95% CI = -0.168, -0.100), FEV_1_/FVC (β = -0.062, 95% CI = -0.082, -0.042) and PEF (β = -0.952, 95% CI = -1.169, -0.735).

**Conclusions:**

Exposure to glyphosate during glyphosate application had significant effects on oxidative stress and lung function in maize farmers.

## Background

Thailand, as an agricultural country and one of the world's largest food exporters, relies significantly on pesticides to protect crops and boost harvests, especially herbicides. The volume of herbicide imported was the highest during the years 2017–2020. The highest imported herbicide was glyphosate [1, 2]. Glyphosate is a weak organic acid of which the formulaic consistency is unclear, often because adjuvants are added to it to make it more effective at killing weeds. In general, it is composed of an isopropylamine salt and a surfactant that is toxic to humans [3, 4]. Glyphosate can enter the body through the skin, respiratory system, and digestive system. Primary exposure in farmers is through the skin and respiratory system while mixing and spraying the herbicides and cleaning equipment. It is absorbed through the cell membrane and enters the blood stream, eventually spreading to the tissues of organs before it is excreted from the body. Some components of glyphosate are excreted through defecation, while some are eliminated from the body by the kidneys through urination which usually occurs within 48 h following exposure [5–7]. Previous cross-sectional studies in farmers found that the use of glyphosate was linked to the onset of various illnesses, including those affecting the respiratory system [8–10]. Laboratory studies added weight to those findings as it was also found that glyphosate has a toxic effect on human lung tissue [11]. However, studies regarding the effects of glyphosate exposure on lung function in agricultural use are still scarce, although indications from some previous laboratory studies showed that exposure to glyphosate caused adverse biological effects such as oxidative stress [12–14].

Oxidative stress is an imbalance between oxidants and anti-oxidants that can impact the human body by damaging cells and tissues, leading to inflammation [15–17]. A previous study found that farmers who are exposed to pesticides experience oxidative stress and increased levels of inflammation [18], although no studies appear to have been carried out investigating the incidence of both conditions among farmers using glyphosate. Based on past research findings, we hypothesized that exposure to glyphosate induces oxidative stress, inflammation, and abnormalities of lung function.

As a result of the review of current findings, the objectives of this study are: (1) to compare urinary glyphosate levels, oxidative stress, inflammation, and lung function before and after applying glyphosate; (2) to identify the factors affecting the increase of urinary glyphosate levels after applying glyphosate in maize farmers; and (3) to investigate the effects of exposure to glyphosate on oxidative stress, inflammation, and lung function after glyphosate application.

## Methods

### Study design and study population

The design of this study is a longitudinal pre-post study. This study design can control invariant (person-specific) confounding factors. Information from questionnaires, urine and blood samples, and lung function performance were collected two days apart, one day before and one day after glyphosate application. Long district, Phrae province, is an area for maize cultivation in northern Thailand, where glyphosate as the major herbicide used. During March and April every year, farmers do not use and are hence not exposed to any pesticides due to it being the post-harvest season. They start to cultivate the maize crop during May and June in every year, therefore, this study was conducted during that time in 2020. The inclusion criteria were: 1) working as a maize farmer in Long District, Phrae Province; 2) apply glyphosate on their farm; and 3) signed a consent form to participate in the study. Farmers who used pesticides for one month before the study and used other pesticides throughout the study were excluded. The sample size for this study was calculated using n4study version 1.4.1, with alpha values of 0.05 and beta values of 0.2. The 180 samples from the calculation result in a statistical power equal to 93.2%. All samples from farmers who had already enrolled for surveys were selected using a simple random sampling approach. Out of 1,356 farmers in the study area, 443 (32.7%) fulfilled the criteria, and 197 (44.5%) agreed to participate in the study. One hundred and eighty were the study subjects with a response rate of 40.6%. This study was approved by the Institutional Review Board on Research Involving Human Subjects of the Faculty of Medicine, Chiang Mai University (no.332/2019, 1 October 2019).

### Interviews

During data collection, the individuals were interviewed face-to-face by public health officials already trained by the researchers. The time taken for the interview was 20 min per person. The collected data included: (1) demographic data (age, gender, education, body mass index (BMI), smoking status, alcohol consumption status, and chronic disease); and (2) agricultural information (distance between the house and the maize farm, spraying equipment, quantities of chemicals used, equipment used in application, role, and personal protective equipment (PPE) use). The interview questionnaire was adapted from the Chiang Mai Lung Health Study interview form [19], which was developed based on the European Community Respiratory Health Survey [20]. This instrument was tested for reliability prior to implementation and the Cronbach’s alpha coefficient was 0.87, indicating that the questionnaire was classed as reliable.

### Urine collection

Urine samples were collected from all participants throughout the 24-h period before and after the application of glyphosate. During collection, urine samples were stored inside foam boxes containing ice until transfer to the laboratory. In the laboratory, urine samples were mixed, divided into 30–50 ml (mL) samples, and frozen at -20 °C until analysis within 2 months.

### Blood collection

Ten mL blood samples were collected on the day before and the day after glyphosate application in the morning, and put into serum separator tubes. The samples were centrifuged at 3,000 revolutions per minute (rpm) for 15 min, and 1.5 mL serum samples were put into sterile Eppendorf tubes, and then refrigerated at -20 °C until analysis within 2 months.

### Measurement of urinary glyphosate levels

The analytical technique described by Jaikwang et al. was used for glyphosate analysis [21] using liquid chromatography-tandem mass spectrometry (LC–MS/MS). The system used was the Agilent 1290 Infinity high-performance liquid chromatography system coupled with an Agilent 6460 triple quadrupole mass spectrometer and electrospray ionization (Agilent Technologies, Inc., Palo Alto, CA, USA). Briefly, a Gemini C6-Phenyl analytical column was used for chromatographic separation, with a gradient elution of 15 mmol per liter of heptafluorobutyric acid in water and acetonitrile. The sample was made by mixing a 100 µl (µl) of an internal standards solution in water (containing 50 µg per liter (µg/L) of 1,2-13C215N glyphosate). Before being injected into the LC–MS/MS, the mixture was filtered using a 0.2 µm (m) nylon membrane filter. Quality control samples with concentrations of 15, 50, and 150 ug/L were used to ensure the analysis was accurate and precise. The accuracy and precision were between 86–105%. The analytical limit of quantification (LOQ) of this method was 5 g/L, with a 2.5 g/L limit of detection (LOD) [21]. The samples with concentrations less than LOD were given the value LOD/square root 2 [22]. Glyphosate levels in the urine were adjusted against urinary creatinine and reported as µg/g creatinine. The urine creatinine values were calculated using the Cobas 8000 analyzer (c701) at Maharaj Nakorn Chiang Mai Hospital Central Laboratory, Faculty of Medicine, Chiang Mai University.

### Analysis of oxidative stress and C-reactive Protein (CRP)

Oxidative stress was determined by modifying the method described by Leelarugrayub et al. [23, 24]. In brief, the level of malondialdehyde (MDA), an intermediate compound of lipid peroxidation, in the serum was measured using modified thiobarbituric acid (TBA). Trichloroacetic acid was used to precipitate 100 µl of serum, which was then combined with 450 µl of normal saline solution (0.9%) and 200 µl of TBA solution. After 30 min in a 90 °C water bath, the entire combination was cooled with water. The absorbance was measured at 532 nm (nm) after centrifugation at 3,500 rpm for 10 min. The concentration of malondialdehyde was estimated from 0–20 micromolar (µM) of standard malondialdehyde **(**Sigma-Aldrich, St. Louis, MO, USA).

The glutathione (GHS) in the serum was measured using the dithiobisnitrobenzoic acid (DTNB) reagent [25]. 3 mL of precipitating solution (0.2 g EDTA, 1.67 g meta-phosphoric acid, and 30 g sodium chloride in 100 mL of distilled water) and 1.6 mL of distilled water were used to precipitate 400 µl of serum and then left to settle for 10 min. This was followed by centrifugation at 3,000 rpm for 5 min. After that, 40 µl of the clear supernatant were collected by suction and mixed with 20 µl of phosphate buffer and 20 µl of DTNB solution. Then the color was measured at 412 nm of absorption. In order to estimate the concentration of glutathione, the samples were compared to a reduced glutathione standard (Sigma-Aldrich, St. Louis, MO, USA). The intra-assay CV is the difference between data points inside an assay and on the same plate. For all MDA and GHS standard concentrations, the coefficient of variation (%CV) ranged from 0.00–7.51 for the pre-sample plate and 0.00–7.11 for the post-sample plate. The linearity of the standard curve had to be more than 0.99 for MDA and GHS to be satisfactory.

The analysis of CRP was measured by nephelometry using the Atellica® NEPH 630 at Maharaj Nakorn Chiang Mai Hospital Central Laboratory, Faculty of Medicine, Chiang Mai University. The LOD of the assay is 0.15 mg/L.

### Measurement of lung function

Participants were tested using a spirometer (CHESTGRAPH HI-105) on the day before and the day after glyphosate application in the morning by a technician following the recommendations of Brian et al. [26]. Before the measurement, the calibration was completed. The following spirometric parameters were recorded for analysis: forced expiratory volume in 1 s (FEV_1_), forced vital capacity (FVC), FEV_1_/FVC, peak expiratory flow (PEF), and forced expiratory flow 25–75% (FEF_25-75%_). Then the best values from the tests were selected.

### Data analysis

Descriptive statistics were used to present frequency distribution, percentage (%), mean, standard deviation (SD), median, 25th percentile (P^25th^), and 75th percentile (P^75th^). Due to the non-normal distributions of glyphosate, MDA, GHS, CRP, FEV1, FVC, FEV1/FVC, PEF, and FEF25-75%, the Wilcoxon matched pairs signed ranked test was used for the comparison of urinary glyphosate levels, oxidative stress, inflammation, and lung function before and after glyphosate application. Multiple linear regression analysis was also used to analyze the factors affecting urinary glyphosate levels after application of glyphosate by maize farmers and the effects of exposure to glyphosate on oxidative stress, inflammation, and lung function after glyphosate application. Due to the mean differences of glyphosate and MDA having a positively skewed distribution and the mean differences of CRP and GHS having a negatively skewed distribution, they were logarithmically transformed before analysis. The potential covariates (univariate analysis *p* < 0.2) were included for the multiple regression model. The covariates for urinary glyphosate level included age, gender, education, spraying equipment, type of spray handle, length of spray handle, the distance between the house and the maize farm, amount of glyphosate, and intensity level of exposure. The covariates for oxidative stress and inflammation included age, gender, education, BMI, smoking status, alcohol consumption, co-morbidities, and urinary glyphosate level. The covariates for lung function included age, gender, education, BMI, smoking status, respiratory diseases, and urinary glyphosate level. The regression analyses were carried out using the entry selection method. Inferential statistics were presented as beta (β), 95% confidence interval (95%CI).

The calculation of the intensity level of exposure was carried out as proposed by Dosemeci et al. [27] using the following formula:

$$Intensity\ level\ of\ exposure = (mixing\ status + application\ method + repair\ status) x\ personal\ protective\ equipment$$

The scores of each parameter were as follows: 0–9 for mixing status, 0–9 for application method, 0–2 for repair status and 0.1–1.0 for personal protective equipment [27].

## Results

The farmers had a mean age of 51.7 ± 8.8 years and a mean BMI of 24 ± 3.9 kg/meters^2^. A small majority of the farmers were male (56.1%), a larger majority smoked (88.3%), 58.9% did not consume alcohol, and 56.1% did not have any chronic diseases. The median distance from home to maize fields was 2 km (P^25th^-P^75th^ = 1.8–2.3). Herbicide application was carried out by the majority of farmers using pump sprayers (96.1%) with normal pressure handles (96.7%). The median amount of glyphosate used was 600 L/day (P^25th^—P^75th^ = 400–1,000), and the median intensity level of exposure was 9.6 (P^25th −^ P^75th^ = 4.8–14.4) (Table 1).

**Table 1:** Demographic characteristics and agricultural information of maize farmers (*N* = 180)

**Parameters**		**Results**
Age (mean ± SD)		51.7 ± 8.8
Gender n (%)	Male	101 (56.1)
	Female	79 (43.9)
Education n (%)	Primary school or lower	129 (71.7)
	Secondary school or higher	51 (28.3)
BMI (kg/m^2^), (mean ± SD)		24 ± 3.9
Smoking status n (%)	Yes	21 (11.7)
	No	159 (88.3)
Alcohol consumption	Yes	74 (41.1)
	No	106 (58.9)
Co-morbidities n (%)	No	101 (56.1)
	Yes	79 (43.9)
	Respiratory diseases	31 (39.2)
	Other co-morbidities	57 (72.2)
Distance between the house and agricultural area (km), median (P^25th^-P^75th^)		2 (1.8–2.3)
Type of herbicide spraying equipment	Backpack	7 (3.9)
	Pump	173 (96.1)
Type of spray handle	High pressure	6 (3.3)
	Normal pressure	174 (96.7)
Length of spray handle (cm), median (P^25th^-P^75th^)		82 (82–82)
Duration of glyphosate application (years), median (P^25th^-P^75th^)		12 (10–20)
Exposure time (hours/day), median (P^25th^-P^75th^)		3 (2–5)
Amounts of glyphosate use (liters/day), median (P^25th^-P^75th^)		600 (400–1,000)
Intensity levels of exposure, median (P^25th^-P^75th^)^a^		9.6 (4.8–14.4)

^a^The intensity level of exposure was calculated as proposed by Dosemeci et al. using the following formula: intensity level of exposure = (mix status + application method + repair status) x personal protective equipment[27]

**Fig. 1:**
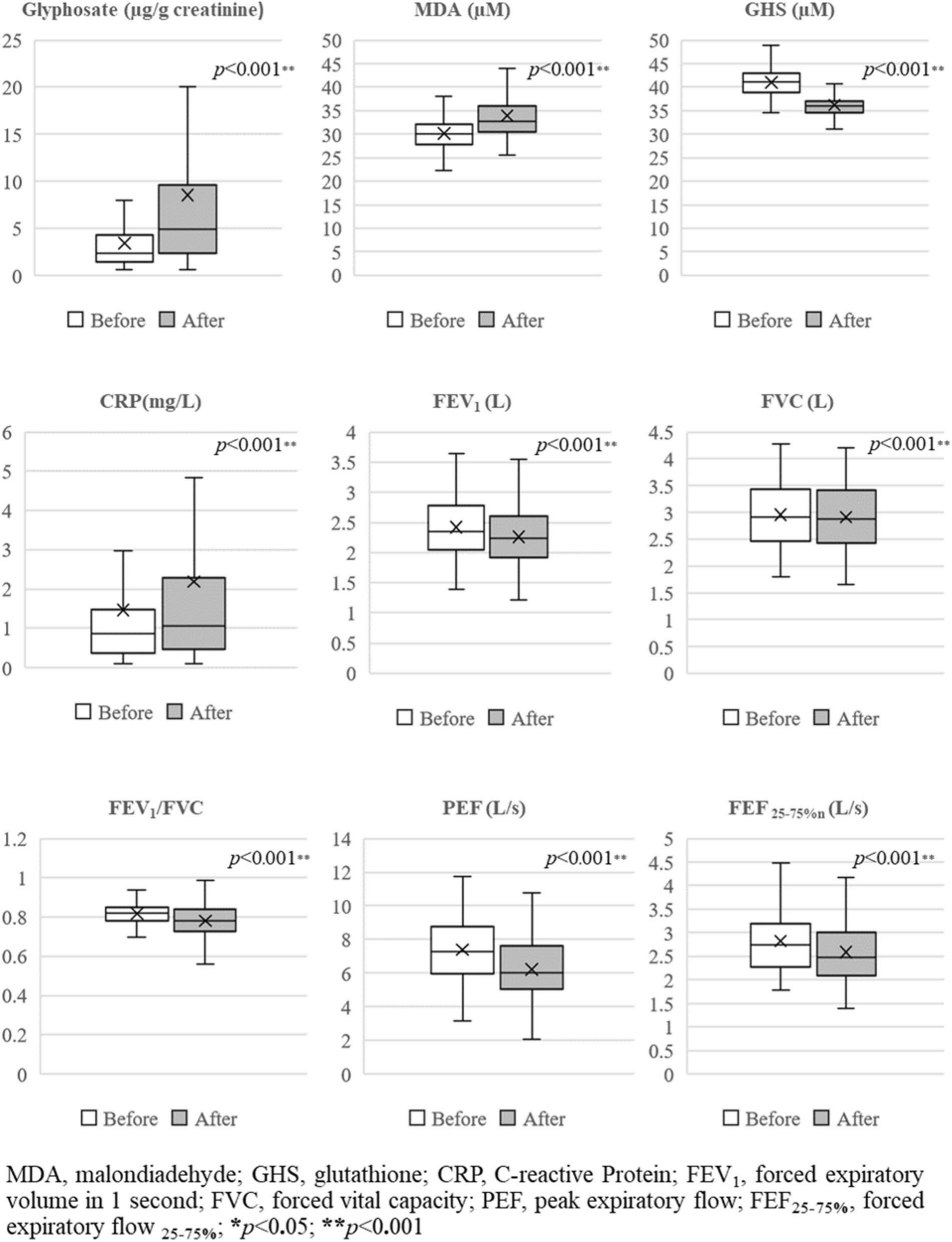
Urinary glyphosate levels, oxidative stress, inflammation, and lung function before and after glyphosate application (*N* = 180)

The comparison of urinary glyphosate levels, oxidative stress, inflammation, and lung function before and after applying glyphosate showed that there was a statistically significant increase in urinary glyphosate levels, oxidative stress and serum MDA (*p* < 0.001), while serum GHS levels showed a statistically significant (*p* < 0.001) decrease. There was a statistically significant increase in inflammation and CRP (*p* < 0.001), however lung function decreased statistically significantly (*p* < 0.001) (Fig. 1).

Multiple linear regression analysis found that the factors contributing to increased urinary glyphosate levels included amount of glyphosate used (β = 0.001, 95% CI = 0.000, 0.001) and intensity level of exposure (β = 0.044, 95% CI = 0.024, 0.063) (Table 2).

**Table 2:** Factors affecting the increase of urinary glyphosate levels after glyphosate application on maize farms (*N* = 180)

Factors	The increase of urinary glyphosate levels	
	**β**	**95% CI**
Age (year)	-0.005	-0.013, 0.003
Gender (male vs female (ref.))	0.004	-0.135, 0.143
Education (primary or lower vs junior high school or higher (ref.))	-0.027	-0.174, 0.119
Spraying equipment (backpack vs pump(ref.))	0.115	-0.204, 0.434
Type of spray handle (high pressure vs normal pressure (ref.))	-0.143	-0.652, 0.365
Length of spray handle (cm)	0.017	-0.012, 0.045
Distance between the house and the maize farm (km)	-0.030	-0.088, 0.028
Amounts of glyphosate used (liter/day)	0.001	0.000, 0.001**
Intensity level of exposure	0.044	0.024, 0.063**

*Β* Beta, *95% CI* 95% confidence interval. *p < 0.05; **p < 0.01

Regarding the effects of exposure to glyphosate on oxidative stress and inflammation after glyphosate application, it was found that urinary glyphosate levels contributed to statistically significant increases in serum MDA (β = 0.024, 95% CI = 0.000, 0.047) and contributed to a statistically significant decrease in serum GHS (β = -0.022, 95% CI = -0.037, -0.007) (Table 3).

**Table 3:** Effects of exposure to glyphosate on oxidative stress and inflammation after glyphosate application (*N* = 180)

Factors	Increased MDA		Decreased GHS		Increased CRP	
	**β**	**95% CI**	**β**	**95% CI**	**β**	**95% CI**
Age (years)	-0.002	-0.004, -0.000*	0.001	-0.001, 0.001	-0.002	-0.011, 0.007
Gender (male vs female (ref.))	-0.017	-0.061, 0.027	0.002	-0.026, 0.030	0.105	-0.111, 0.320
Education (primary school vs secondary school or higher (ref.))	0.017	-0.011, 0.045	-0.008	-0.026, 0.010	0.167	0.030, 0.305*
BMI (kg/m^2^)	-0.004	-0.007, -0.001*	0.000	-0.002, 0.002	0.024	0.008, 0.040**
Smoking status (yes vs no (ref.))	0.015	-0.023, 0.053	0.002	-0.022, 0.026	0.075	-0.110, 0.260
Alcohol consumption (yes vs no (ref.))	-0.005	-0.048, 0.039	-0.008	-0.036, 0.020	-0.048	-0.260, 0.164
Co-morbidities (yes vs no (ref.))	-0.002	-0.025, 0.022	0.005	-0.010, 0.019	0.133	0.020, 0.250*
Urinary glyphosate levels (μg/g creatinine)	0.024	0.000, 0.047*	-0.022	-0.037, -0.007**	0.044	-0.069, 0.157

*MDA* Malondialdehyde, *GHS* Glutathione, *CRP* C-reactive Protein, *β* Beta, *95% CI* 95% confidence interval. *p < 0.05; **p < 0.01

With regard to the effects of exposure to glyphosate on lung function after glyphosate application, it was found that urinary glyphosate levels contributed to statistically significantly decreased FEV_1_ (β = -0.134, 95% CI = -0.168, -0.100), FEV_1_/FVC (β = -0.062, 95% CI = -0.082, -0.042) and PEF (β = -0.952, 95% CI = -1.169, -0.735) (Table 4).

**Table 4:** Effects of exposure to glyphosate on lung function after glyphosate application (*N* = 180)

Factors	FEV_1_		FVC		FEV_1_/FVC		PEF		FEF_25-75%_	
	**β**	**95% CI**	**β**	**95% CI**	**β**	**95% CI**	**β**	**95% CI**	**β**	**95% CI**
Age (year)	-0.000	-0.003, 0.002	0.000	-0.003, 0.003	-0.001	-0.002, 0.000	0.006	-0.008, 0.020	-0.003	-0.009, 0.002
Gender (male vs female (ref.))	-0.087	-0.125, -0.049**	-0.004	-0.060, 0.052	-0.022	-0.044, 0.000	-0.565	-0.805, -0.325**	-0.065	-0.159, 0.030
Education (primary school vs secondary school or higher (ref.))	-0.000	-0.042, 0.041	0.004	-0.058, 0.065	-0.013	-0.037, 0.011	-0.054	-0.317, 0.208	0.068	-0.036, 0.171
BMI (kg/m^2^)	0.002	-0.003, 0.007	-0.005	-0.012, 0.002	0.003	-0.000, 0.005	0.015	-0.016, 0.045	-0.005	-0.017, 0.007
Smoking status (yes vs no (ref.))	0.028	-0.028, 0.084	-0.016	-0.099, 0.067	0.032	-0.001, 0.065	0.052	-0.303, 0.407	-0.025	-0.165, 0.116
Respiratory diseases (yes vs no (ref.))	-0.035	-0.081, 0.010	0.013	-0.054, 0.080	-0.019	-0.045, 0.007	-0.147	-0.435, 0.141	-0.045	-0.158, 0.069
Urinary glyphosate levels (μg/g creatinine)	-0.134	-0.168, -0.100**	0.009	-0.042, 0.059	-0.062	-0.082, -0.042**	-0.952	-1.169, -0.735**	-0.015	-0.100, 0.071

*FEV*_*1*_ Forced expiratory volume in 1 s, *FVC* Forced vital capacity, *PEF* Peak expiratory flow, *FEF*_*25-75%*_ Forced expiratory flow 25–75%, *β* Beta, *95% CI* 95% confidence interval. *p < 0.05; **p < 0.01

## Discussion

Our results found that urinary glyphosate levels increased after the act of applying glyphosate. This finding is consistent with previous studies [28, 29]. Glyphosate is a herbicide composed of several chemicals, including isopropylamine salt and a surfactant that enhances the herbicidal effectiveness of the glyphosate. Glyphosate can enter the body through breathing, the skin, and the eyes [3, 30], and occupational exposure in farmers can occur when they mix, apply, and clean their equipment [6]. Glyphosate can be excreted through the urinary system without any changes in its chemical structure having a biological half-life in humans of approximately 3 ½ to 14 ½ hours [31, 32]. Therefore, measurement of glyphosate in urine can be used as a biomarker of glyphosate exposure [27]. Previous studies also suggested that urinary glyphosate levels contributed to the amount, duration, frequency, and the intensity level of glyphosate exposure [33]. Lack of or incorrect use of PPE has also been shown to affect urinary glyphosate levels [34–37].

In the case of serum oxidative stress and inflammation, our results indicated that serum MDA and CRP levels increased statistically significantly after the application of the glyphosate, but that GHS decreased. These findings are consistent with a previous study carried out in Algeria which found that farmers who were exposed to pesticides had higher MDA and CRP levels and lower GHS levels (*p* < 0.001 for MDA; *p* < 0.01 for GHS) [18]. Similarly, a study in India comparing people who were exposed to pesticides through spraying and unexposed controls found that sprayers had higher MDA levels than the unexposed group (*p* < 0.001) [37], possibly due to the toxic mechanism of the surfactant in the glyphosate.

Since surfactants can penetrate the walls of mitochondria and destroy the proton gradients essential for energy production, a loss of homeostatic balance and increased oxidative stress occur, and a state of imbalance develops between oxidants and anti-oxidants causing excessive production of free radicals [3, 17]. Free radicals can react with most cellular molecules, including lipids and proteins. Previous studies found that exposure to glyphosate increased lipid peroxidase activity by 130% and reduced glutathione-s-transferase action by 70–80%. Oxidative damage occurs when oxygen-derived free radicals attack the double bonds in unsaturated fatty acids found in membrane lipids, producing various lipid peroxidation products. Among the many different products that can be formed as secondary products during lipid peroxidation, MDA is one [17, 38, 39].

When a cell is damaged by oxidative stress, it has a defense mechanism that produces antioxidants to destroy excess free radicals [40, 41]. GHS is an antioxidant compound with a sulfhydryl group (-SH) in its molecule which is found in almost every cell, playing a vital role in many cell processes, such as protecting cells from damage from oxidative stress [42]. In vivo, oxidative stress caused by glyphosate is caused by a decrease in glutathione and an increase in the products of lipid peroxidation. The loss of glutathione comes from this antioxidant breaking down glyphosate through the activity of GHS-peroxidase [43].

CRP is a marker of inflammation, which increases after tissue injury. CRP causes enhanced monocyte activation, adhesion, and transmigration, as well as causing the generation of reactive oxygen species and activation of complement, all critical pathophysiological variables associated with tissue injury [44, 45]. In previous studies, an increase in CRP in farmers using pesticides was found [18], however, our results found no association between urinary glyphosate levels and CRP levels.

Lung function, measured using FEV_1_, FVC, FEV_1_/FVC, PEF, and FEF_25-75%_, decreased statistically significantly after the application of glyphosate. This finding is consistent with a study carried out in South Korea which found that farmers who used paraquat herbicide had decreased FVC and FEV_1_ (β = -5.20, *p* < 0.001 for FVC; β = -1.89, *p* = 0.010 for FEV_1_) [46]. These findings also concur with a previous study in Thailand which found that the values of FVC%, FEV_1_%, and PEFR% after spraying pesticides were statistically significantly lower than before spraying pesticides (*p* = 0.012 for FVC%; *p* = 0.02 for FEV_1_%; *p* = 0.022 for PEFR%) [47]. Similarly, a study in India found that the value of FEV_1_ after spraying of pesticides was statistically significantly lower than before spraying of pesticides (*p* < 0.05) [35]. This might have been due to the lack of use of PPE and / or incorrect use of PPE causing pesticides to be able to enter the body during application or after application in farmers present on farm land [34, 48]. Inhalation into the lungs is a typical mechanism for pesticides to enter the body. Exposure to pesticides has been linked to an increase in lung dysfunction in pesticide applicators [11, 48]. Glyphosate, whose toxicity has been shown in both in vitro and in vivo studies to affect inflammation in lung and airway tissues, has also been shown to cause higher amounts of eosinophils, neutrophils, and asthma-related cytokines (IL-5, IL-10, IL-13, IL-33, TSLP), which result in narrowing of the airway [11, 48, 49]. In addition, the small pesticide vapors can affect the efficiency of the alveolar gas exchange, making it less effective [34, 48].

In summary, this study evaluated various biomarkers before and after the application of glyphosate to indicate any causal relationships. Even though the study had clear inclusion criteria and used multiple linear regression analysis, there were several limitations. Firstly, oxidative stress is non-specific biomarker. The effects of other variables on oxidative stress and inflammation included the impact of ultraviolet (UV) rays and the use of dietary supplements. It is not possible to make firm conclusions based on an increase that is observed after the use of glyphosate without referring to what happens independently from the use of glyphosate. However the findings from this study warrant further investigation in this very important area with a focus on minimizing the impact of confounding variables. Secondly, although a longitudinal pre-post study can control control invariant (person-specific) confounding factors, it can not clearly explain the effects of glyphosate exposure. Therefore, the comparison the effects between the farmers who are exposed and not exposed to glyphosate should be investigated further. Finally, this study investigated the effects of acute exposure; therefore, the effects of long-tern-exposure should be investigated further.

## Conclusions

Exposure to glyphosate had a negative impact on oxidative stress and lung function in farmers who applied glyphosate resulting in an increase in serum MDA and a decrease in serum GHS, FEV_1_, FEV_1_/FVC, and PEF. Further studies to assess the long-term effects of glyphosate are warranted.

## Data Availability

The data used in this study can be made available from the authors on reasonable request.

## Abbreviations

MDA: Malondialdehyde
GHS: Glutathione
CRP: C-reactive protein
FEV_1_: Forced expiratory volume in 1 second
FVC: Forced vital capacity
PEF: Peak expiratory flow
FEF _25__–__75__%_: Forced expiratory flow 25–75%
BMI: Body mass index
PPE: Personal protective equipment
rpm: Revolutions per minute
mL: Milliliter
µl: Microliter
µg/L: Micrograms per liter
µg/g: Micrograms per gram
µM: Micromolar
mg/L: Milligrams per liter
L: Liter
L/s: Liters per second
LC–MS/MS: Liquid chromatography-tandem mass spectrometry
LOQ: Limit of quantification
LOD: Limit of detection
TBA: Thiobarbituric acid
DTNB: Dithiobisnitrobenzoic acid
nm: Nanometer
km: Kilometer
cm: Centimeter
SD: Standard deviation
β: Beta
SE.: Standard error

## References

[1] Panuwet P, Siriwong W, Prapamontol T, Ryan PB, Fiedler N, Robson MG (2012). Agricultural Pesticide Management in Thailand: Situation and Population Health Risk. Environ Sci Policy.

[2] Department of Agriculture. Available online: https://www.doa.go.th/ard/?page_id=386 (Accessed on 25 May 2023).

[3] Bradberry SM, Proudfoot AT, Vale JA (2004). Glyphosate poisoning. Toxicol Rev.

[4] Williams GM, Kroes R, Munro IC (2000). Safety evaluation and risk assessment of the herbicide Roundup and its active ingredient, glyphosate, for humans. Regul Toxicol Pharmacol.

[5] European Food Safety Authority (2015). Conclusion on the Peer Review of the pesticide risk assessment of the active substance glyphosate. EFSA J.

[6] Harvey B (2014). Protecting Farming Families & Field-Workers. Prof Saf.

[7] Acquavella JF, Alexander BH, Mandel JS, Gustin C, Baker B, Chapman P (2004). Glyphosate biomonitoring for farmers and their families: Results from the farm family exposure study. Environ Health Perspect.

[8] Hoppin JA, Umbach DM, Long S, London SJ, Henneberger PK, Blair A (2017). Pesticides are Associated with Allergic and Non-Allergic Wheeze among Male Farmers. Environ Health Perspect.

[9] Hoppin JA, Umbach DM, London SJ, Henneberger PK, Kullman GJ, Alavanja MCR (2008). Pesticides and atopic and nonatopic asthma among farm women in the agricultural health study. Am J Respir Crit Care Med.

[10] Hoppin JA, Umbach DM, London SJ, Lynch CF, Alavanja MCR, Sandler DP (2006). Pesticides associated with wheeze among commercial pesticide applicators in the agricultural health study. Am J Epidemiol.

[11] Hao Y, Zhang Y, Ni H, Gao J, Yang Y, Xu W (2019). Evaluation of the cytotoxic effects of glyphosate herbicides in human liver, lung, and nerve. J Environ Sci Heal Part B, Pestic food Contam Agric wastes..

[12] Tarouco F, Godoi F, Velasques R, Guerreiro A, Geihs M, Rosa C (2017). Effects of the herbicide Roundup on the polychaeta Laeonereis acuta: Cholinesterases and oxidative stress. Ecotoxicol Environ Saf.

[13] Sinhorin VDG, Sinhorin AP, Miléski KML, Hansen PC, Moreira PSA, S Teixeira JM dos (2014). Effects of the acute exposition to glyphosate-based herbicide on oxidative stress parameters and antioxidant responses in a hybrid Amazon fish surubim (Pseudoplatystoma sp). Ecotoxicol Environ Saf.

[14] Nwani CD, Nagpure NS, Kumar R, Kushwaha B, Lakra WS (2013). DNA damage and oxidative stress modulatory effects of glyphosate-based herbicide in freshwater fish. Channa Punctatus Environ Toxicol Pharmacol.

[15] Kehrer JP, Klotz LO (2015). Free radicals and related reactive species as mediators of tissue injury and disease: implications for Health. Crit Rev Toxicol.

[16] Spickett CM, Pitt AR (2012). Protein oxidation: role in signalling and detection by mass spectrometry. Amino Acids.

[17] Sailaja Rao P, Kalva S, Yerramilli A, Mamidi S (2011). Free Radicals and Tissue Damage: Role of Antioxidants. Free Radicals Antioxidants.

[18] Madani FZ, Hafida M, Merzouk SA, Loukidi B, Taouli K, Narce M (2016). Hemostatic, inflammatory, and oxidative markers in pesticide user farmers. Biomarkers.

[19] Pothirat C, Phetsuk N, Liwsrisakun C, Bumroongkit C, Deesomchok A, Theerakittikul T (2016). Major chronic respiratory diseases in Chiang Mai: Prevalence, clinical characteristics, and their correlations. J Med Assoc Thail.

[20] Burney PGJ, Luczynska C, Chinn S, Jarvis D (1994). The European Community Respiratory Health Survey. Eur Respir J.

[21] Jaikwang P, Junkuy A, Sapbamrer R, Seesen M, Khacha-ananda S, Mueangkhiao P (2020). A Dilute-and-Shoot LC–MS/MS Method for Urinary Glyphosate and AMPA. Chromatographia.

[22] Hornung RW, Reed LD (1988). Estimation ofaverage concentration in the presence of nondetectable values. Appl Occup Environ Hyg.

[23] Leelarungrayub N, Ketsuwan N, Pothongsunun P, Klaphajone J, Bloomer RJ (2011). Effects of N-acetylcysteine on oxidative stress, interleukin-2, and running time in sedentary men. Gazz Medica Ital Arch per le Sci Mediche..

[24] Leelarungrayub N, Sutabhaha T, Pothongsunun P, Chanarat N (2005). Exhaustive exercise test and oxidative stress response in althetic and sedentary subject. CMU J.

[25] Beutler E, Duron O, Kelly BM (1963). Improved method for the determination of blood glutathione. J Lab Clin Med.

[26] Graham BL, Steenbruggen I, Miller MR, Barjaktarevic IZ, Cooper BG, Hall GL (2019). Standardization of Spirometry 2019 Update An Official American Thoracic Society and European Respiratory Society Technical Statement. Am J Respir Crit Care Med.

[27] Dosemeci M, Alavanja MCR, Rowland AS, Mage D, Zahm SH, Rothman N (2002). A quantitative approach for estimating exposure to pesticides in the agricultural health study. Ann Occup Hyg.

[28] Zhang F, Xu Y, Liu X, Pan L, Ding E, Dou J, et al. Concentration distribution and analysis of urinary Glyphosate and its metabolites in occupationally exposed workers in eastern china. Int J Environ Res Public Health. 2020;17(8). 10.3390/ijerph17082943 10.3390/ijerph17082943 PMC7215609 32344631

[29] Connolly A, Jones K, Galea KS, Basinas I, Kenny L, McGowan P (2017). Exposure assessment using human biomonitoring for glyphosate and fluroxypyr users in amenity horticulture. Int J Hyg Environ Health.

[30] Gandhi K, Khan S, Patrikar M, Markad A, Kumar N, Choudhari A (2021). Exposure risk and environmental impacts of glyphosate: Highlights on the toxicity of herbicide co-formulants. Environ Challenges.

[31] Connolly A, Jones K, Basinas I, Galea KS, Kenny L, McGowan P (2019). Exploring the half-life of glyphosate in human urine samples. Int J Hyg Environ Health.

[32] Myers JP, Antoniou MN, Blumberg B, Carroll L, Colborn T, Everett LG (2016). Concerns over use of glyphosate-based herbicides and risks associated with exposures: A consensus statement. Environ Heal A Glob Access Sci Source..

[33] National Pesticide Information Center. Available online: http://npic.orst.edu/factsheets/archive/glyphotech.html (Accessed on 5 Oct 2021).

[34] Fareed M, Pathak MK, Bihari V, Kamal R, Srivastava AK, Kesavachandran CN (2013). Adverse Respiratory Health and Hematological Alterations among Agricultural Workers Occupationally Exposed to Organophosphate Pesticides: A Cross-Sectional Study in North India. PLoS ONE.

[35] Pathak MK, Fareed M, Srivastava AK, Pangtey BS, Bihari V, Kuddus M (2013). Seasonal variations in cholinesterase activity, nerve conduction velocity and lung function among sprayers exposed to mixture of pesticides. Environ Sci Pollut Res.

[36] Chakraborty S, Mukherjee S, Roychoudhury S, Siddique S, Lahiri T, Ray MR (2009). Chronic exposures to cholinesterase-inhibiting pesticides adversely affect respiratory health of agricultural workers in India. J Occup Health.

[37] Kesavachandran C, Singh VK, Mathur N, Rastogi SK, Siddiqui MKJ, Reddy MMK (2006). Possible mechanism of pesticide toxicity-related oxidative stress leading to airway narrowing. Redox Rep.

[38] Samanta P, Pal S, Mukherjee AK, Ghosh AR (2014). Biochemical effects of glyphosate based herbicide, Excel Mera 71 on enzyme activities of acetylcholinesterase (AChE), lipid peroxidation (LPO), catalase (CAT), glutathione-S-transferase (GST) and protein content on teleostean fishes. Ecotoxicol Environ Saf.

[39] Ayala A, Muñoz MF, Argüelles S. Lipid peroxidation: Production, metabolism, and signaling mechanisms of malondialdehyde and 4-hydroxy-2-nonenal. Oxid Med Cell Longev. 2014;2014. 10.1155/2014/360438 10.1155/2014/360438 PMC4066722 24999379

[40] Pham-Huy LA, He H, Pham-Huy C (2008). Free radicals, antioxidants in disease and health. Int J Biomed Sci.

[41] Young IS, Woodside JV (2001). Antioxidants in health and disease. J Clin Pathol.

[42] Gaucher C, Boudier A, Bonetti J, Clarot I, Leroy P, Parent M. Glutathione: Antioxidant properties dedicated to nanotechnologies. Antioxidants. 2018;7(5). 10.3390/antiox7050062 10.3390/antiox7050062 PMC5981248 29702624

[43] El-Shenawy NS (2009). Oxidative stress responses of rats exposed to Roundup and its active ingredient glyphosate. Environ Toxicol Pharmacol.

[44] Thiele JR, Zeller J, Kiefer J, Braig D, Kreuzaler S, Lenz Y, et al. A conformational change in C-reactive protein enhances leukocyte recruitment and reactive oxygen species generation in ischemia/reperfusion injury. Front Immunol. 2018;9(APR). 10.3389/fimmu.2018.00675 10.3389/fimmu.2018.00675 PMC5911593 29713320

[45] Eisenhardt SU, Habersberger J, Murphy A, Chen YC, Woollard KJ, Bassler N (2009). Dissociation of pentameric to monomeric C-reactive protein on activated platelets localizes inflammation to atherosclerotic plaques. Circ Res.

[46] Cha ES, Lee YK, Moon EK, Kim YB, Lee YJ, Jeong WC (2012). Paraquat application and respiratory health effects among South Korean farmers. Occup Environ Med.

[47] Sapbamrer R, Thongtip S, Khacha-ananda S, Sittitoon N, Wunnapuk K (2020). Changes in lung function and respiratory symptoms during pesticide spraying season among male sprayers. Arch Environ Occup Heal.

[48] Hernández AF, Casado I, Pena G, Gil F, Villanueva E, Pla A (2008). Low level of exposure to pesticides leads to lung dysfunction in occupationally exposed subjects. Inhal Toxicol.

[49] Kumar S, Khodoun M, Kettleson EM, McKnight C, Reponen T, Grinshpun SA (2014). Glyphosate-rich air samples induce IL-33, TSLP and generate IL-13 dependent airway inflammation. Toxicology.

